# Melanin Synthesis Inhibition Activity of Compounds Isolated from Bamboo Shoot Skin (*Phyllostachys pubescens*)

**DOI:** 10.3390/molecules28010023

**Published:** 2022-12-20

**Authors:** Ahmed Ashour, Ahmed Elbermawi, Yhiya Amen, Ahmed E. Allam, Hiromi Ikeda, Maki Nagata, Kenta Kumagae, Tomoyo Azuma, Aya Taguchi, Takuya Takemoto, Masako Matsumoto, Kuniyoshi Shimizu

**Affiliations:** 1 Department of Pharmacognosy, Faculty of Pharmacy, Prince Sattam Bin Abdulaziz University, Al-Kharj 11942, Saudi Arabia; 2 Department of Pharmacognosy, Faculty of Pharmacy, Mansoura University, Mansoura 35516, Egypt; 3 Department of Agro-Environmental Sciences, Graduate School of Bioresource and Bioenvironmental Sciences, Kyushu University, 744 Motooka, Nishi-ku, Fukuoka 819-0395, Japan; 4 Department of Pharmacognosy, Faculty of Pharmacy, Al-Azhar University, Assiut 71524, Egypt; 5 Faculty of Agriculture, Setsunan University, 45-1 Nagaotoge-cho, Osaka 573-0101, Japan; 6 Sansho Pharmaceutical Co., Ltd., 2-26-7 Ohike, Fukuoka 816-8550, Japan

**Keywords:** bamboo shoot skin, melanin inhibition, *Phyllostachys pubescens*, agro-waste

## Abstract

This study targets the evaluation of melanin synthesis inhibition activity of the bamboo shoot skin as agro-waste. The total methanolic extract of bamboo peel extract was evaluated for its skin protective effects via measuring its melanin inhibitory activity and its suppression activity on the expression of tyrosinase mRNA levels. Results showed that bamboo peel extract has a good ability for the inhibition of melanin synthesis so further studies were performed for the isolation of its constituents. Twelve compounds have been isolated from the shoot skin of *Phyllostachys pubescens*. Their structures were elucidated based on extensive spectroscopic methods. The melanin inhibition potential of the isolates was tested with their collagen-production-promoting activity for the determination of active principles. Results showed that Betulinic acid, tachioside, and 1,2-dilinolenin significantly suppressed melanin production per cell compared to control. Triacontanol, tricin, and (+)-lyoniresinol 9′-*O*-glucoside also tended to decrease melanin production per cell. These findings indicated that the skin of bamboo shoots, a significant agricultural waste, is a useful natural source for further research on its potential for aging problems such hyperpigmentation and cognitive function impairment.

## 1. Introduction

In our continuous search for material for skin disorders [1,2], phytochemical and biological investigation of different fractions and isolated compounds from bamboo shoot skin (*Phyllostachys pubescens*) was carried out.

In Japan, *Phyllostachys pubescens* (*P. pubescens*) is a widely prevalent species. Since the dawn of civilization, one of the most significant and precious renewable resources has been utilized by people [3].

Skin hyperpigmentation is one of the common skin conditions that can affect people with all different types of skin. Hyperpigmentation is the term used to describe the increased production and distribution of melanin, the pigment that gives human skin its color [4]. Many human populations experience some types of hyperpigmentation, including post-inflammatory hyperpigmentation, age spots, solar lentigo, and melisma, which are regarded as skin disorders.

Several studies have reported on antioxidant, anticancer, and antibiotic properties [5,6]. In these studies, various active compounds were isolated from leaves such as flavones and their glycosylated form, whose aglycones are represented by apigenin, luteolin, tricin, glycosides, in addition to phenolic acids, coumarin lactones, anthraquinones, and amino acids [7,8,9,10,11,12,13].

Numerous investigations have been carried out to prove *P. pubescens’* biological activity. Most of these studies have focused on extracts from specific *P. pubescens* organs. For instance, the anti-inflammatory, anti-allergic, and anti-cancer characteristics of the leaves and branches [14], as well as the antibacterial capabilities of the stems, shoots, and shoot skins [15,16,17].

A preparation known as an antioxidant of bamboo leaves (AOB), which is applied as a food antioxidant and whose usage is approved by the local Health Ministry is made in China using the phenolic components of *P. pubescens*. Lactones, phenolic acids, and flavonoids make up the majority of the AOB. The flavone C-glycosides orientin, homoorientin, vitexin, and isovitexin are the major flavonoids present in AOB [18].

In previous studies in our laboratory, anti-allergy and anti-bacterial activities of extracts from different parts of *P*. *pubescens* were investigated [19,20]. The study of natural plant products has led to the identification of many potentially useful chemicals that could be used as new depigmenting treatments with fewer negative effects [21,22,23].

In this study, we isolated some of the active compounds related to skin activities such as melanin, hyaluronic acid, and collagen production in a way that found whitening compounds, retained moisture, and protected from wrinkle effects.

## 2. Results and Discussion

### 2.1. The Effect of Bamboo Peel Extract on Melanin Production

To estimate the effect of bamboo peel extract on melanin production, we measured melanin production in mouse melanoma cells and in a three-dimensional human skin model after application of bamboo peel extract.

**Figure 1:**
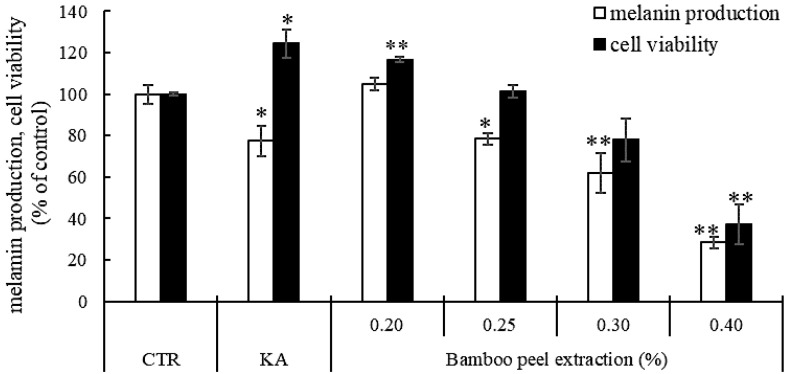
Inhibitory effects of different concentrations of bamboo peel extract on melanin synthesis in B16 melanoma cells (* *p* < 0.05 and ** *p* < 0.01).

As a result, bamboo peel extracts decreased melanin production in melanoma cells compared to control (Figure 1). In particular, 0.25% bamboo peel extract increased the cell viability by more than 100% and inhibited melanin production until 78.4% compared to control. Although, 0.4% bamboo peel extract also suppressed the amount of melanin production in mouse melanoma cells, which was due to reduced cell viability.

**Figure 2:**
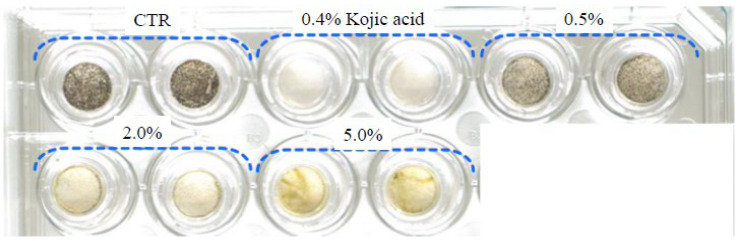
Photograph of three-dimensional human skin model (CTR stands for Control).

Next, we evaluated melanin production after the application of bamboo peel extract in a three-dimensional human skin model. Figure 2 shows an appearance photograph of this three-dimensional human skin model after application of the bamboo peel extract. In this result, the bamboo peel extract inhibited melanin production in a dose-dependent manner.

**Figure 3:**
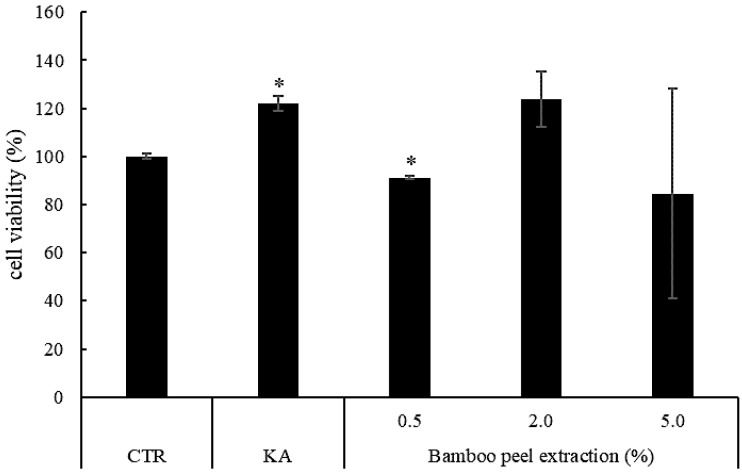
Cell viability of different concentrations of bamboo peel extract on melanin synthesis in three-dimensional human skin model (CTR—abbreviation of Control, KA—Kojic acid, * *p* < 0.05).

However, 5.0% of the bamboo peel extract inhibited the amount of melanin production, while a green precipitate was observed. In the application of 2.0% bamboo peel extract, cell viability was increased compared to control (Figure 3). On the other hand, 0.5% and 5.0% of the bamboo peel extract decreased cell viability, however it was more than 80%. These results suggest that the bamboo peel extract has an inhibitory effect on melanin production in the three-dimensional human skin model.

### 2.2. Effect of the Bamboo Peel Extract on Tyrosinase

**Figure 4:**
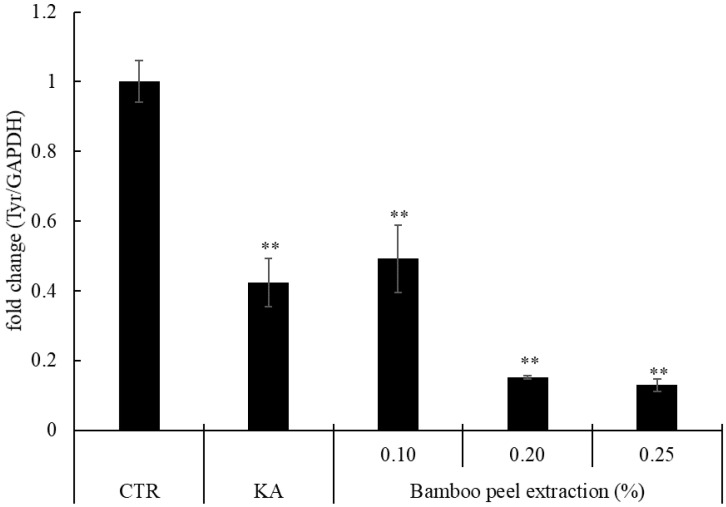
Relative amounts of tyrosinase gene expression after application of bamboo peel extract (** *p* < 0.01).

We confirmed that the bamboo peel extract decreased melanin production in melanoma cells and in the three-dimensional human skin model. To estimate the tyrosinase activity, which is involved in melanin production, we examined tyrosinase mRNA levels by using a real-time PCR analysis. Melanoma cells were exposed to the bamboo peel extract in a concentration 0.1, 0.2, and 0.25% for 96 h and the tyrosinase mRNA level in B16 cells were measured. As a result, the expression of tyrosinase mRNA level by bamboo peel extract was the same and/or decreased compared to kojic acid as a positive control. Notably, the treatment of 0.25% bamboo peel extract decreased at maximum 87.1% of the expression of tyrosinase mRNA level than control (Figure 4).

We found that the bamboo peel extract suppressed the expression of tyrosinase mRNA levels. To estimate the effect of the bamboo peel extract on the accumulation of tyrosinase protein in B16 cells, we detected tyrosinase protein using western blot analysis. Figure 4 shows the accumulation of tyrosinase protein after adding the bamboo peel extract. By treating the bamboo peel extract, the amounts of tyrosinase protein were less than the control. The result indicated that the bamboo peel extract had the suppression activity of tyrosinase protein production.

### 2.3. Chemical Isolation

**Figure 5:**
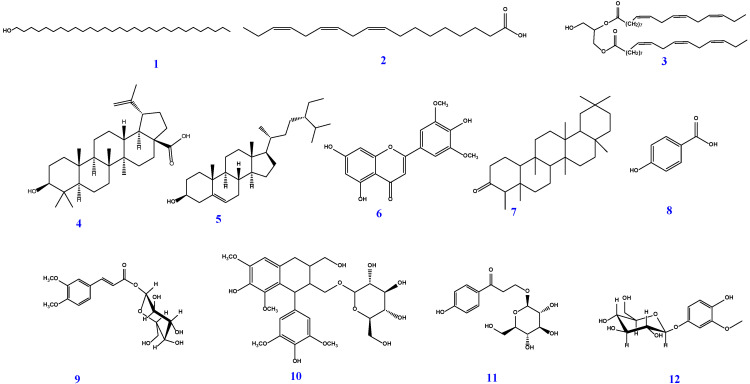
Chemical structures of compounds 1–12, isolated from peels of *P. pubescens*.

From these results, there is a possibility that the bamboo peel extract contains compounds which have skin whitening effects by suppressing melanin production. We isolated several compounds from the bamboo peel and then identified this. Chemical investigation of different fractions of bamboo shoot skin led to the isolation of 12 compounds (Figure 5): triacontanol (1) [24], 1,2-dilinolenin (2) [25,26], linolenic acid (3), betulinic acid (4) [24], *β*-sitosterol (5) [27], tricin (6), friedelin (7) [28], 4-hydroxybenzoic acid (8) [29], 9-*O*-*β*-d-glucopyranosyl-3,4-dimethoxy-cinnamic acid (9) [30], (+)-lyoniresinol 9′-*O*-glucoside (10), 3,4′-dihydroxypropiophenone 3-*O*-glucoside (11), and tachioside (12) [31].

All compounds were identified following analysis of their spectroscopic data (1D- and 2D-NMR), and in comparison with previously published reports.

### 2.4. Effects of Bamboo Isolated Compounds on Production of Melanin, Hyaluronic Acid, and Collagen

To estimate the function of isolated compounds from *P. pubescens* peel against human skin, we examined the effect of isolated compounds from *P. pubescens* peel on production of melanin, hyaluronic acid, and collagen.

**Figure 6:**
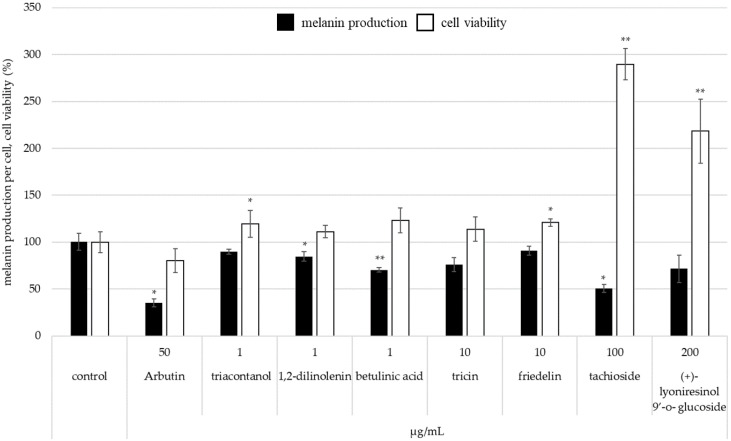
The inhibitory effect of isolated compounds from bamboo peel extract on melanin synthesis in B16 melanoma cell (* *p* < 0.05 and ** *p* < 0.01).

Figure 6 shows melanin production per cell after addition of each isolated compound to B16 melanoma cell. Betulinic acid, tachioside, and 1,2-dilinolenin significantly suppressed melanin production per cell compared to control.

Triacontanol, tricin, and (+)-lyoniresinol 9′-ο-glucoside also tended to decrease melanin production per cell (Figure 6). In addition, 1,2-dilinolenin, betulinic acid, and tricin did not have an effect on cell viability. In the case of application of tachioside or (+)-lyoniresinol 9′-*O*-glucoside, cell viability was higher than control (Figure 6). Thus, each isolated compound from *P. pubescens* skin has a suppression effect on melanin production because of increasing cell viability.

**Figure 7:**
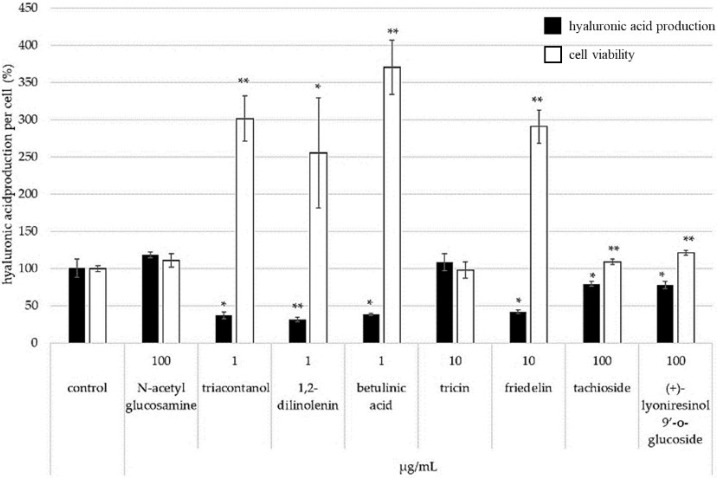
Effect of isolated compounds on the production of hyaluronic acid (* *p* < 0.05 and ** *p* < 0.01).

Figure 7 shows hyaluronic acid per cell after application of each isolated compound. Tricin has an effect to increase hyaluronic acid production per cell. On the other hand, triacontanol, 1,2-dilinolenin, betulinic acid, and friedelin were lower in production of hyaluronic acid per cell than control because cell viability was higher.

**Figure 8:**
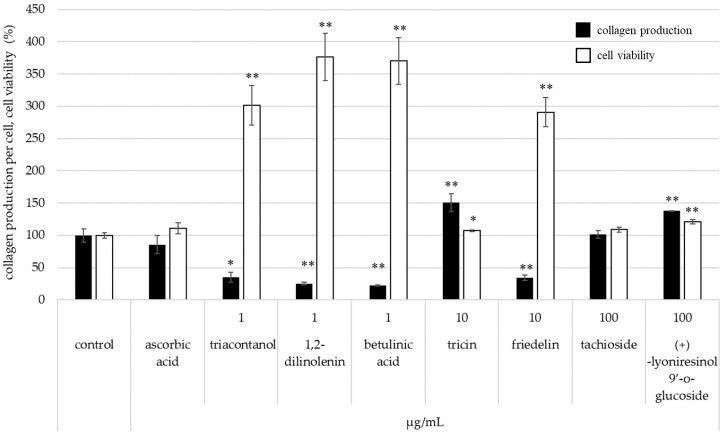
Effect of isolated compounds on the production of collagen (* *p* < 0.05 and ** *p* < 0.01).

Figure 8 shows collagen production per cell after treatment of each isolated compound. Tricin and (+)-lyoniresinol 9′-*O*-glucoside have an effect to increase collagen production. These results indicated that isolated compounds of bamboo affect skin whitening, retain moisture, and protect wrinkles.

In particular, tricin has the potential for skin moisture retention, elasticity, and wrinkle protection by increasing production of hyaluronic acid and collagen.

## 3. Material and Methods

### 3.1. General Experimental Procedures

Organic solvents and Silica gel (75–120 mesh) for open column chromatography (CC) were purchased from Wako Pure Chemical Industries (Osaka, Japan). Analytical NP-TLC (silica gel 60 GF_254_ plates, 20 × 20 cm × 0.2 mm thick) and RP-TLC (RP-C_18_ F_254_ plates, 5 × 7.5 cm × 0.2 mm thick), both obtained from Merck (Darmstadt, Germany). TLC plates were envisioned under UV light (SLUV-6, AS One, Osaka, Japan) at 254 and 365 nm and then heated at 105 °C for 5 min after being sprayed with 5% sulfuric acid in methanol. Preparative RP-TLC Silica gel 60 RP-18 F_254_S glass plates were obtained from Merck (Darmstadt, Germany) and Preparative NP-TLC Silica gel 70 FM TLC glass plates (Wako Industrial Company, Shimizu, Japan) were used for final purification of the compounds. One-dimensional and two-dimensional NMR experiments were carried out on a Bruker DRX 600 NMR spectrometer (Bruker Daltonics Inc., Billerica, MA, USA), using TMS as an internal standard at 600.00 MHz for ^1^H-NMR and 150.9 MHz for ^13^C-NMR. Chemical shifts (δ) were expressed in ppm, while the coupling constant (*J*) in Hz.

### 3.2. Waste Material

The peels of *Phyllostachys pubescens* were used in this study. They were provided from Kazue Bussan Co. Ltd. (Fukuoka, Japan).

### 3.3. Preparation of Plant Extracts

The peel of *P. pubescens* were air-dried and ground to a fine powder. The powder (2.7 kg) was extracted by maceration using methanol (MeOH) at room temperature (3 × 5 L, each 48 h). The combined extracts were concentrated under vacuum at 40 °C to give 183 g of dried extract. The MeOH extract (approximately 180 g) was dissolved in a least amount of MeOH/H_2_O in a separating funnel and then, using liquid–liquid fractionation, consecutively extracted with *n*-hexane, ethyl acetate (EtOAC), and finally, with *n*-butanol (*n*-BuOH). The obtained organic phases, in each case, were evaporated at 45 °C to dryness under low pressure using a rotary evaporator to afford 60.0, 40.0 and 45.0 g, respectively, of *n*-hexane (H), EtOAC (E), and *n*-BuOH (B) fractions. The extracts were stored at 4 °C for chemical investigation.

### 3.4. Isolation and Purification

About 55 g of hexane extract (H) was fractionated over a silica gel Column Chromatography (CC) using a gradient elution of *n*-hexane–EtOAc (100:0→0:100) to give 41 sub-fractions. Sub-fraction 1 was purified by crystallization to afford one compound; 1 (10 mg). Sub-fractions (14–16) were chromatographed over a silica gel column using a gradient elution of *n*-hexane–EtOAc (100:0→80:20) to give three pure compounds; 2 (3.7 mg), 3 (4.4 mg), and 4 (3.2 mg). Sub-fractions 19–22 were purified by crystallization to afford one compound; 5 (12 mg). Sub-fractions (31–35) were initially separated using preparative normal phase TLC (P NP-TLC) on (Silica gel 70 FM TLC glass plates) using *n*-hexane–EtOAc (70:30), to give one compound, which was further purified by several crystallizations using CH_2_Cl_2_-MeOH to give compound 6 (15 mg).

The EtOAc extract (38 g) was subjected to a silica gel CC eluted with *n*-hexane–EtOAc (100:0→0:100), then EtOAc–MeOH (100:0→40:60) to finally give 20 sub-fractions. Sub-fractions 1–4 were purified by crystallization using CH_2_Cl_2_–MeOH (3:1) accompanied by evaporation of CH_2_Cl_2_ in the air to furnish compound 7 (100 mg). Sub-fractions (14–16) were purified using preparative normal phase TLC (PNP-TLC) on (Silica gel 70 FM TLC glass plates) using *n*-hexane–EtOAc (10:90) to give one pure compound: 8 (2.7 mg).

The aqueous extract (43 g) was subjected to a silica gel CC eluted with EtOAc–MeOH (100:0→0:100) to finally give 18 sub-fractions. Sub-fraction 16 was applied onto the top of RP-C18 column using isocratic elution of H_2_O-MeOH (50:50) to give one pure compound: 9 (5.7 mg). Sub-fractions 17–18 were purified using P RP-TLC using H_2_O-MeOH (60:40) to give three pure compounds: 10 (3.6 mg), 11 (3.5 mg), and 12 (1.2 mg).

### 3.5. Melanin Assay for Bamboo Peel Extract

#### 3.5.1. Preparation of Bamboo Peel Extract

For all bioassays, except three bioassays which used the isolated compounds from the bamboo peel, the bamboo peel extract prepared by the following method was used.

We collected and crushed the green bamboo stalk of skin (particle size 4 mm). An amount of 6.4 kg of green bamboo stalk of skin was mixed with 64 kg of ethanol. The static extraction took place in room temperature for 48 h. The sample was then filtered, and the filtrate was collected. Compound 1, 3-butylene glycol (BG) was added to the residue, and ethanol was distilled off by concentration under reduced pressure (70 °C). The concentrated liquid was filtered to prepare a bamboo skin extract.

#### 3.5.2. Cell Culture

Mouse melanoma (B16-F10) cells were obtained from ATCC (Manassas, VA, USA). B16-F10 cells were incubated and maintained in Eagle’s Minimum Essential Media (EMEM) with 10% fetal bovine serum (FBS). The cells were incubated in a 24-well plate at a density of 0.017 × 10^5^ cells/well in a humidified atmosphere containing 5% CO_2_ at 37 °C.

#### 3.5.3. Determination of Melanin Production

B16-F10 cells were incubated in a 24-well plate at a density of 0.017 × 10^5^ cells/well. After 48 h, added samples (0.2, 0.225, 0.25, 0.275, 0.3, and 0.4% of bamboo peel extract) or solvent as a control (EMEM with 10% FBS) to the cells. Kojic acid (2.5 mM) was used as a positive control. The cells were incubated for 72 h. After incubation, the cells were washed by PBS (-) 2 times and 2 mol/L NaOH was added to the cells for dissolution at 65 °C for 1 h. After dissolution, absorbance at 470 nm wavelength was measured with the microplate reader (Biotek, Winooski, VT, USA).

#### 3.5.4. Cell Viability Assay

The cell viability of B16-F10 cells was used by Cell Count Reagent SF (Nacalai tesque, Kyoto, Japan). After incubation, the supernatant was collected, and the solution (EMEM and Cell Count Reagent SF) added for 1.5 h. After incubation, the absorbance at 450 nm wavelength was measured with the microplate reader.

### 3.6. Melanin Assay for Three-Dimensional Human Skin Model

#### 3.6.1. Cell Culture

Three-dimensional human skin model (MEL-312B kit, Black donor) was obtained from KURABO (Osaka, Japan). The human skin model was maintained according to the manufacturer’s instructions. The human skin model was placed in 6-well plates and incubated for 1 h at 37 °C in 5% CO_2_. An amount of 0.1 mL of sample solution (0.5, 2, and 5% of bamboo peel extract) were added into the model cup surface. PBS was used as a control and 0.4% kojic acid in ultra-pure water was used as a positive control. The tissue was incubated for 14 days and fed with 5 mL of fresh EPI-100LLMM (KURABO, Osaka, Japan) every 2 or 3 days. On day 14, the tissue surface was washed 2 times with PBS and added 0.3 mL/well in 24-well plate.

#### 3.6.2. MTT Assay

The MTT assay was used to determine the tissue cells’ vitality after 14 days of incubation. The human skin model was put into a 24-well plate, and each well received 0.3 mL of MTT solution (MTT diluted with medium: 1 mg/mL). The tissue was then incubated at 37 °C in 5% CO_2_ for 3 h. After incubation, the surface was collected into 1.5 mL tube. An amount of 500 μL of MTT extraction solution (isopropanol) was added to each tube and incubated in a cool dark place for 42 h. After incubation, each tube was centrifuged at 10,000 rpm, 5 min, and the supernatant transferred to 200 μL/well in a 96-well plate. The absorbance of extracts was measured at 570–650 nm.

### 3.7. Melanin, Hyaluronic Acid, and Collagen Assay for Bamboo Isolated Compounds

#### 3.7.1. Cell Culture

Mouse melanoma (B16) cells and Normal Human Dermal Fibroblast-adult (NHDF-Ad) cells were obtained from the Riken Bioresource Center (Ibaraki, Japan). In Dulbecco’s Modified Eagle Medium (DMEM) low glucose accompanied with 10% fetal bovine serum (FBS) and 1% penicillin-streptomycin (PS) solution, B16 cells were incubated and kept alive. NHDF cells were maintained in DMEM high glucose with 10% FBS and 1% PS solution. The cells were incubated in a humidified atmosphere containing 5% CO_2_ at 37 °C.

#### 3.7.2. Determination of Melanin Production

B16 cells were incubated in 24-well plate at a density of 0.5 × 10^5^ cells/well. After 24 h, samples or solvent were added (Water or DMSO) to the cells. Arbutin was used as a positive control. The cells were incubated for 24 h. After incubation, the medium was replaced to trypsin EDTA solution to collect the cells into the tubes. The cells were washed with medium and PBS. After that, dissolved with 1N NaOH and incubated at 100 °C for 10 min and measured the absorbance at 450 nm wavelength with the microplate reader.

#### 3.7.3. Cell Viability Assay

The cell viability of B16 cells was used by Cell counting kit-F (Dojindo Molecular Technologies, Kumamoto, Japan). After incubation, the supernatant was collected and the solution (DMEM and Cell counting kit-F) added for 3 h. After incubation, the absorbance at 450 nm wavelength was measured with the microplate reader.

#### 3.7.4. Measurement of Hyaluronic Acid and Collagen Production

Seed NHDF cells into 96-well plate at a density of 0.5 × 10^5^ cells/100 µL/well with DMEM supplemented with 10% FBS and 1% PS. Twenty-four hours later, replace the medium with DMEM supplemented with 1% PS. Added the samples or solvent (Water or DMSO) to the cells. Ascorbic acid is used as positive control for collagen. The cells were incubated at 37 °C, 5% CO_2_. Three days later, collect supernatant to a new 96-well plate and keep it in −30 °C refrigerator until assay.

The hyaluronic acid and collagen ELISA assay followed the manual exactly using QnE Hyaluronic acid ELISA assay (Biotech Trading Partners Inc., Encinitas, CA, USA) and Human Collagen 1 ELISA kit (ACEL, Tokyo, Japan), respectively.

#### 3.7.5. Cell Viability Assay

The remaining cells were washed with DMEM containing 1% SP for one time. Adding DMEM containing 1% SP and 5 mg/mL MTT (100 µL/well) to the cells. After incubating the cells at 37 °C containing 5% CO_2_ for 4 h, formazan crystals were dissolved in the cells with isopropanol-HCl solution (100 µL/well) and the absorbance at 570 nm wavelength was measured with the microplate reader.

### 3.8. Real-Time PCR

The B16-F10 cells were seeded in 100-mm dishes at 0.4 × 10^5^ cells per dish. After 24 h of incubation, bamboo peel extract or solvent as a control (EMEM with 10% FBS) was added to the B16 cells. Kojic acid (2.5 mM) was used as a positive control. The B16 cells were then cultured for 96 h. Total RNA was purified from the cultured B16 cells using ISOGENE II (Nippon Gene, Tokyo, Japan). The cDNA strand was synthesized from 500 ng of total RNA using ReverTra Ace qPCR RT Master Mix (TOYOBO, Osaka, Japan). The quantitative real-time PCR was performed in a total reaction volume of 25 µL using SYBR Premix Ex Taq II (TaKaRa Bio, Shiga, Japan) and 20 ng cDNA per reaction. Real-time PCR was performed using Thermal Cycler Dice Real Time System Lite TP700 (TakaRa, Japan) under the following conditions: 30 s at 95 °C, followed by 40 cycles each of 95 °C for 5 s, 60 °C for 30 s. Primers used for amplification were as follows: GTCGTCACCCTGAAAATCCTAACT and CATCGCATAAAACCTGATGGC for Tyrosinase, TTGTGCAGTGCCAGCCTC and CCAATACGGCCAAATCCG for GAPDH.

### 3.9. Western Blot Analysis

The B16-F10 cells were cultured with bamboo peel extract for 96 h as already described. After the medium had been removed, the cells were washed twice with PBS and lysed in cell lysis solution (50 mM Tris-HCl, 150 mM NaCl, 0.1% SDS, 1% Sodium Deoxycholate, 1% TritonX-100, 1 × protein inhibitor). Each cell lysate was centrifuged at 14,000 rpm for 10 min at 4 °C, and the supernatant was collected. After the protein was measured, 13.4 µg of total protein was applied by SDS-PAGE on acrylamide gel and then transferred to a PVDF membrane. After transfer, the membrane was washed three times in TBS-T (20 mM Tris, 150 mM NaCl, 0.05% Polyoxyethylene Sorbitan Monolaurate) for 10 min. The membrane was blocked in TBS containing 3% skim milk for 1 h and subsequently washed twice in TBS-T for 5 min. The membrane was then incubated with primary antibodies (Anti-tyrosinase) in TBS containing 1% skim milk overnight at 5 °C and subsequently washed three times in TBS-T for 10 min. Next, the membrane was then incubated in TBS containing 1% skim milk and secondary antibodies (Anti-mouse IgG) for 1 h at room temperature and then washed three times in TBS-T for 10 min. For reprobing, blotted membrane was treated with stripping buffer (0.4 M Glycine, 0.2% SDS, 2% Polyoxyethylene Sorbitan Monolaurate) for 1 h at room temperature. The membrane was washed three times with TBS-T and then blocked for 1 h at room temperature with TBS containing 3% skim milk. The membrane was subjected to antibody (primary antibodies: Anti-β-Actin, secondary antibodies: Anti-mouse IgG) reactions as described above.

## 4. Conclusions

Phytochemical and biological research of various fractions and isolated chemicals from bamboo shoot skin was conducted as part of our ongoing search for handling skin problems. The expression of tyrosinase mRNA by bamboo peel extract was either the same as or lower when compared to kojic acid, a positive control. Meanwhile, melanin synthesis was drastically inhibited in cancer cells and a three-dimensional model of human skin. Furthermore, results from a western blot study demonstrated that bamboo peel extract has the power to prevent the production of the tyrosinase protein. So far, the production of melanin, hyaluronic acid, and collagen were also examined in relation to isolated compounds from *P. pubescens* peel; betulinic acid, tachioside, and 1,2-dilinolenin dramatically reduced melanin production per cell compared to the control. In addition to increasing collagen production, tricin and (+)-lyoniresinol 9’-O-glucoside also boost hyaluronic acid production per cell. The results suggest that bamboo skin peels are a promising natural source for further studies on the development of new medications, which might be useful in aging disorders such as declining of cognitive functions and hyperpigmentation.

## Data Availability

Not applicable.

## Sample Availability

Samples of the compounds are available from the authors.
